# Molecular analysis of the endobronchial stent microbial biofilm reveals bacterial communities that associate with stent material and frequent fungal constituents

**DOI:** 10.1371/journal.pone.0217306

**Published:** 2019-05-29

**Authors:** John E. McGinniss, Ize Imai, Aurea Simon-Soro, Melanie C. Brown, Vincent R. Knecht, Laura Frye, Priyanka M. Ravindran, Marisol I. Dothard, Dylan A. Wadell, Michael B. Sohn, Hongzhe Li, Jason D. Christie, Joshua M. Diamond, Andrew R. Haas, Anthony R. Lanfranco, David M. DiBardino, Frederic D. Bushman, Ronald G. Collman

**Affiliations:** 1 Department of Medicine, Division of Pulmonary, Allergy and Critical Care, Perelman School of Medicine at the University of Pennsylvania, Philadelphia, Pennsylvania, United States of America; 2 Department of Epidemiology, Biostatistics and Informatics, Perelman School of Medicine at the University of Pennsylvania, Philadelphia, Pennsylvania, United States of America; 3 Department of Microbiology, Perelman School of Medicine at the University of Pennsylvania, Philadelphia, Pennsylvania, United States of America; Wadsworth Center, UNITED STATES

## Abstract

Endobronchial stents are increasingly used to treat airway complications in multiple conditions including lung transplantation but little is known about the biofilms that form on these devices. We applied deep sequencing to profile luminal biofilms of 46 endobronchial stents removed from 20 subjects primarily with lung transplantation-associated airway compromise. Microbial communities were analyzed by bacterial 16S rRNA and fungal ITS marker gene sequencing. *Corynebacterium* was the most common bacterial taxa across biofilm communities. Clustering analysis revealed three bacterial biofilm types: one low diversity and dominated by *Corynebacterium*; another was polymicrobial and characterized by *Staphylococcus*; and the third was polymicrobial and associated with *Pseudomonas*, *Streptococcus*, and *Prevotella*. Biofilm type was significantly correlated with stent material: covered metal with the *Staphylococcus*-type biofilm, silicone with the *Corynebacterium*-dominated biofilm, and uncovered metal with the polymicrobial biofilm. Subjects with sequential stents had frequent transitions between community types. Fungal analysis found *Candida* was most prevalent, *Aspergillus* was common and highly enriched in two of three stents associated with airway anastomotic dehiscence, and fungal taxa not typically considered pathogens were highly enriched in some stents. Thus, molecular analysis revealed a complex and dynamic endobronchial stent biofilm with three bacterial types that associate with stent material, a central role for *Corynebacterium*, and that both expected and unexpected fungi inhabit this unique niche. The current work provides a foundation for studies to investigate the relationship between stent biofilm composition and clinical outcomes, mechanisms of biofilm establishment, and strategies for improved stent technology and use in airway compromise.

## Introduction

Endobronchial stents are frequently implanted to treat airway complications, with lung transplantation being a particularly common setting for indications including dehiscence, stenosis, and malacia [1,2]. Stents are composed of various materials, including uncovered metal, covered metal, and silicone [3]. Despite their value in treating medical conditions, implanted biomaterials typically become colonized via biofilms. In other biomedical devices, biofilms have been linked to device failure, especially in immunosuppressed and critically ill hosts [4]. Bacterial biofilms may also contain potential pathogens, and organisms within biofilms are more resistant to host immunity and antimicrobials than free-floating planktonic forms [5]. Biofilms on central venous catheters, intra-cardiac devices, and orthopedic devices has been explored extensively [4,5]. Understanding the nature of biofilms that form on implanted devices is an essential first step in assessing biofilm impact on outcome and/or optimizing such devices. Remarkably little is known about biofilms on endobronchial stents, however.

Emerging culture-independent molecular tools have revolutionized our ability to understand the composition of microbial communities [6–8]. Such sequence-based approaches can comprehensively define entire communities, are highly quantitative, and do not depend on *a priori* knowledge of suspected constituents or the ability to culture individual organisms. These methods have recently been applied to oral [9–11] and endotracheal tube biofilms[12–16]. Here we present the first sequence-based analysis of luminal biofilms of endobronchial stents used for clinical treatment of critical airway compromise, most of which were in the setting of lung transplant-associated airway complications.

## Materials and methods

### Subjects and sample collection

Endobronchial stents were collected from March 2014 through November 2016 from subjects who were undergoing clinically-indicated bronchoscopic removal due to need for stent replacement or to resolution of the disease process. Stents were initially placed in the airway and subsequently retrieved through rigid bronchoscopy by a team of interventional pulmonary specialists. Following removal, stents were placed in sterile specimen cups containing sterile saline for transport to the lab (15–30 minutes) and then gently rinsed again to dissociate poorly adherent material. The lumen was then vigorously swabbed circumferentially (Copan Diagnostics), and swabs kept at -80°C. In four cases, bronchoalveolar lavage (BAL) was available from the same bronchoscopy. Clinical data was extracted from the electronic medical record. All subjects gave written informed consent under protocols approved by the University of Pennsylvania IRB (protocol #823558, #812748, #817513, #820073).

### Bacterial and fungal amplification, sequencing and analysis

DNA extraction, PCR amplification, and sequencing details are included in supplemental materials. Briefly, DNA was extracted from swabs by combined chemical and mechanical (BioSpec Mini-Beadbeater-16) methods using PowerSoil DNA isolation kit (MoBio) with an additional 95°C incubation to improve DNA recovery from fungi. Extracted DNA was amplified using barcode-labelled primers (27F forward and 338R reverse) directed at the bacterial 16S rRNA gene V1-V2 and fungal ITS-1 regions (ITS1F forward and ITS2 reverse), and sequenced on the Illumina MiSeq platform with 250-bp paired-end reads [17]. ITS amplification was assessed using automated electrophoresis with Agilent 22000 TapeStation system (Agilent Technologies, Santa Clara, CA) and if no detectable amplification occurred, or if the amplification was less than that of primer dimers, these samples were not pooled for sequencing. As a control for environmental background, sterile swabs exposed to saline in collection cups were analyzed in parallel.

Bacterial 16S rRNA gene sequences were clustered into *de novo* operational taxonomic units (OTUs) at 97% sequence similarity using UCLUST in the QIIME 1.91 pipeline [18], and then aligned to the GreenGenes reference database (v13_8) using PyNAST. Samples with more than 1000 reads (which included all stent swab samples) and OTU’s with 2 or more hits across samples were kept for downstream analysis. Fungal ITS sequence data were processed with the PIPITS pipeline, which uses ITSx software that employs hidden Markov models to extract known ITS sequences from the amplicons. This was followed by annotation of 97% percent identity OTU representative sequences with BROCC and manual BLASTn results [19,20]. Bacterial analysis was based on relative abundances, except when comparing BAL with bronchoscopic pre-wash samples where we also examined absolute read counts so that taxa within low microbial biomass pre-wash samples are not exaggerated [6,21]. Fungal analysis was carried out using absolute read numbers rather than relative abundances given the high variability in fungal quantity between samples, also to avoid exaggeration of taxa with high proportions in specimens with very low fungal content [22]. Sequence data are available in the NCBI Short Read Archives (Project SRP154880) and our code and metadata are available at: https://github.com/johnmcginniss/stent/.

### Statistical analysis

All figures and statistical tests were conducted in R (v.3.4.4; http://www.r-project.org). Alpha diversity (richness, Shannon index) was calculated using the *vegan* package. Beta diversity (UniFrac distances) was calculated in the QIIME pipeline (v.1.91; http://qiime.org) and used to perform principal coordinate analysis (PCoA) [23]. Clinical data were summarized using range, median, and interquartile range. Pairwise Wilcoxon rank sum test was used to compare between-group differences with Bonferroni-Holm correction for multiple testing. Fisher’s exact test was used to analyze categorical data. Permutational multivariate analysis of variance (PERMANOVA) with 999 permutations was used to calculate group differences in weighted UniFrac distances. We used the hclust function from the R *stats* package for hierarchical clustering and the pam function from the *cluster* package for partitioning around medoids analysis (PAM) of weighted UniFrac data [24], with further details in the S1 File.

## Results

### Subjects and stent samples

We analyzed the luminal biofilm of 46 airway stents from 20 subjects (Table 1). Nineteen were treated for airway complication following lung transplantation: 3 for anastomotic dehiscence, and 14 for stenosis or dynamic airway collapse (indication was not available for 2 subjects). All transplant subjects were maintained on an immunosuppression regimen of multiple agents (generally tacrolimus, mycophenolate, and prednisone). One subject had non-transplant-associated dynamic airway collapse. Stents were silicone (n = 20), uncovered metal (n = 18) or covered metal (n = 8).

**Table 1:** Subject and stent characteristics.

Subject	Lung Diagnosis^A^	Trans-plant	Stent type	Stent location	Day Post-Transplant^B^	Stent duration (days)	Stent Top Taxa by OTU Count^C^	Concurrent Bronchoscopic Culture^D^	ITS Analysis^E^
**0002**	IPF	Left	Silicone	Left	474	371	1. Anaerococcus (g) 2. Porphyromonas (g)	NA	+
**0018**	COPD	Left	Silicone	Left	301	42	1. Streptococcus (g) 2. Veillonella (g)	NA	+
			Covered	Left	310	9	1. Streptococcus (g) 2. Staphylococcus (g)	NA	+
			Silicone	Left	353	53	1. Streptococcus (g) 2. Actinomyces (g) 10. Pseudomonas (g)	1. *Pseudonomas aeruginosa* 2. Mouth flora	+
			Covered	Left	548	195	1. Nesseriaceae (f) 2. Pseudomonas (g)	NA	+
**0079**	Non-IPF ILD	Left	Silicone	Left	454	348	1. Corynebacterium (g) 2. Anaerococcous (g)	NA	BT
**0720**	CF	BLT	Silicone	Left	785	21	1. Corynbacterium (g) 2. Enterobacteriaceae (f)	NA	+
			Silicone	Right	785	21	1. Corynbacterium (g) 2. Enterobacteriaceae (f)	NA	+
**0777**	COPD	BLT	Uncovered	Right	67	42	1. Prevotella (g) 2. Fusobacterium (g)	NA	+
			Uncovered	Right	98	31	1. Fusobacterium (g) 2. Prevotella (g) 3. Pseudomonas (g)	*1*. *Pseudomonas aeruginosa* 2. Aspergillus spp.	+
**0778**	COPD	BLT	Uncovered	Left	20	4	1. Pseudomonas (g) 2. Ureaplasma (g)	NA	BT
**0877**	IPF	Right	Silicone	Right	330	55	1. Corynebacterium (g) 2. Finegoldia (g)	1. Mouth flora	+
**0895**	IPF	Right	Silicone	Right	157	14	1. Corynebacterium (g) 2. Streptococcus (g)	1. Mouth flora 2. *Mycobacterium avium* intracellulare	+
			Silicone	Right	214	28	1. Corynebacterium (g) 2. Prevotella (g)	NA	BT
**0935**	Non-CF bronchiectasis	BLT	Uncovered	Left	79	23	1. Mycoplasma (g) 2. Streptococcus (g)	NA	+
			Uncovered	Right	79	27	1. Mycoplasma (g) 2. Streptococcus (g)	NA	+
**0937**	COPD	BLT	Silicone	Right	163	72	1. Staphylococcus (g) 2. Streptococcus (g)	NA	+
**0985**	IPF	Left	Silicone	Left	130	8	1. Enterobacteriaceae (f) 2. Streptococcus (g)	NA	+
**0988**	IPF	BLT	Uncovered	Right	51	15	1. Pseudomonas (g) 2. Enterobacteriaceae (f)	NA	+
			Uncovered	Right	72	9	1. Pseudomonas (g) 2. Enterococcus (g)	NA	BT
			Uncovered	Left	85	13	1. Streptococcus (g) 2. Corynebacterium (g)	NA	BT
			Uncovered	Right	85	6	1. Corynebacterium (g) 2. Streptococcus (g)	NA	+
			Uncovered	Left	93	7	1. Streptococcus (g) 2. Corynebacterium (g)	NA	BT
			Uncovered	Left	99	13	1. Pseudomonas (g) 2. Corynebacterium (g)	NA	BT
**0991**	IPF	BLT	Silicone	Right	128	51	1. Corynebacterium (g) 2. Staphylococcus (g)	NA	BT
			Silicone	Left	189	112	1. Prevotella (g) 2. Veillonella (g)	NA	BT
			Covered	Left	247	58	1. Staphylococcus (g) 2. Pseudomonas (g)	NA	BT
			Silicone	Left	317	51	1. Stapylococcus (g) 2. Corynebacterium (g)	NA	+
**1000**	IPF	Left	Uncovered	Left	98	16	1. Prevotella (g) 2. Campylobacter (g) 11. *S*. *aureus* (s)* 32. Enterobacteriacea (f)	1. *Staphylococcus aureus* 2. *Serratia marcescenans*	BT
**8001**	COPD	BLT	Silicone	Left	1932	1455	1. Fusobacterium (g) 2. Corynebacterium (g)	NA	BT
			Silicone	Right	1932	723	1. Corynebacterium (g) 2. Pseudomonas (g)	NA	+
**8002**	COPD (non-transplant)	NA	Silicone	Left	NA	304	1. Corynebacterium (g) 2. Actinomyces (g)	NA	BT
**8003**	COPD	BLT	Silicone	Left	649	436	1. Corynebacterium (g) 2. Anaerococcus (g)	NA	BT
**8004**	IPF	Left	Silicone	Right	979	248	1. Corynebacterium (g) 2. Pseudomonas (g)	NA	BT
**0048**	IPF	Left	Covered	Left	287	87	1. Staphylococcus (g) 2. Peptoniphilus (g)	NA	+
			Covered	Left	475	188	1. Staphylococcus (g) 2. Anaerococcus (g)	1. Mouth flora	+
**0099**	Non-IPF ILD	BLT	Uncovered	Left	143	14	1. Parvimonas (g) 2. Campylobacter (g)	NA	+
			Uncovered	Right	143	14	1. Parvimonas (g) 2. Campylobacter (g)	NA	BT
			Covered	Left	151	8	1. Veillonella (g) 2. Bulleidia (g)	NA	+
			Uncovered	Right	151	8	1. Parvimonas (g) 2. Campylobacter (g)	NA	BT
			Uncovered	Right	199	48	1. Parvimonas (g) 2. Corynebacterium (g)	1. Mouth flora	+
			Covered	Left	253	33	1. Corynebacterium (g) 2. Anaerococcus (g)	NA	BT
			Uncovered	Right	253	33	1. Corynebacterium (g) 2. Anaerococcus (g)	NA	BT
			Covered	Left	262	9	1. Corynebacterium (g) 2. Anaerococcus (g) 3. Enterococcus (g)	1. *Enterococcus faecalis* 2. Mouth flora	+
			Uncovered	Right	262	9	1. Corynebacterium (g) 2. Prevotella (g)	1. *Enterococcus faecalis* 2. Mouth flora	BT
			Silicone	Right	267	5	1. Corynebacterium (g) 2. Enterococcus (g)	1. Mouth flora	BT

^A^Lung Diagnosis is the disease leading to transplantation. One subject (#8002) was a non-transplant subject.

^B^Day post-transplant refers to the number of days post-transplant on which the stent was removed.

^C^For each sample, the OTU-based taxa were collapsed into the lowest taxonomic assignment available in our analysis. These were then ranked on absolute read count. Reported are the top two taxa per sample. If the cultured organism was not in the top two it is reported with its respective rank.

^D^Culture is from bronchoalveolar lavage or tissue. NA denotes no culture taken at the time of stent removal.

^E^+ denotes sample that underwent ITS sequencing; samples without sufficient ITS amplicon for sequencing are indicated as below threshold (BT).

* 2/3 of reads assigned to *Staphylococcus* in this sample were able to be identified as *S*.*aureus* using BLASTn.

### Bacterial 16S rRNA gene sequence analysis of biofilm communities

Stent biofilm samples were subject to bacterial 16S rRNA gene amplification using V1-V2 primers followed by Illumina sequencing. After quality filtering we had approximately 6.88x10^6^ reads in total, with a median read count per stent biofilm sample of 53,471 (IQR 46,252–64,614). After 97% clustering this yielded 13,769 *de novo* OTUs in the swab samples.

The amount of 16S amplicon, as a surrogate for bacterial biomass [22,25], was markedly greater in stent biofilms compared to several types of controls (p<0.001 comparing biofilm samples to laboratory background and bronchoscope prewash; S1 Fig). The S2 Fig shows a heatmap of taxa in stent and control samples.

**Fig 1:**
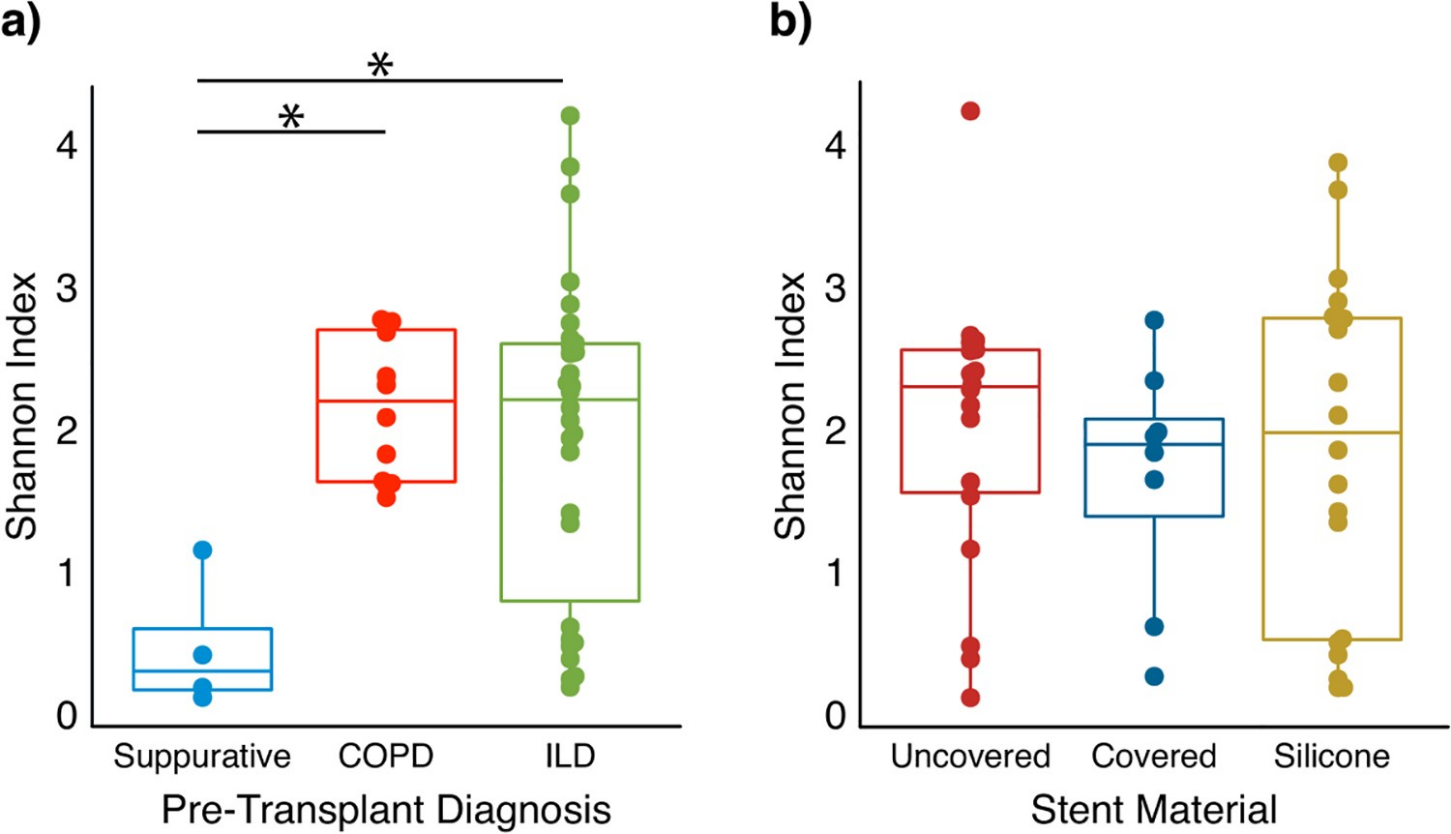
Diversity of stent biofilm bacterial communities by underlying disease and stent material. Biofilm communities were grouped by underlying pulmonary diagnosis (A) and stent material (B), and within-sample bacterial diversity was calculated using the Shannon Index. Each dot represents a sample, the boxplot represents the median, 75th and 25th percentiles, and points outside the whiskers are outliers. Subjects with underlying suppurative lung disease (cystic fibrosis (CF) and non-CF bronchiectasis) had lower alpha diversity compared to COPD and ILD (p < 0.05, pairwise comparison Wilcoxon rank sum test). Stent material did not impact alpha diversity.

Subjects transplanted for suppurative lung disease had lower within-sample diversity (alpha-diversity as measured by the Shannon index) compared to those transplanted for ILD (p = 0.0134) or COPD (p = 0.0033) (Fig 1A). In contrast, alpha-diversity did not differ across the three stent materials (Fig 1B).

**Fig 2:**
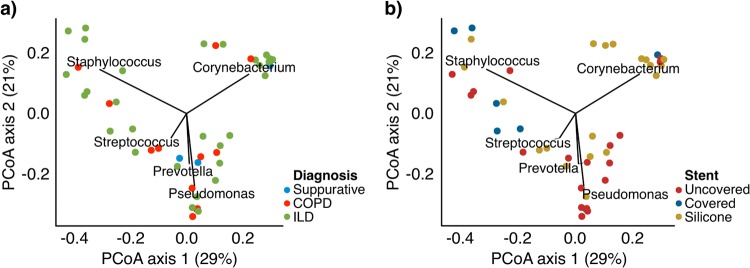
Principal coordinate analysis of stent microbial communities. Biofilm communities were analyzed by Principal Coordinate Analysis (PCoA), using weighted UniFrac. Each sample is represented by a dot. Panel (A) is colored by underlying diagnosis and panel (B) is by stent material. The vectors represent the genus-level bacteria present above 5% mean relative abundance that explain the ordination on the PCoA. The length of the vector is proportional to its explanatory power.

We examined between-sample diversity (beta-diversity) using a principal coordinate analysis (PCoA) of weighted UniFrac distances (Fig 2). UniFrac compares communities based on shared phylogenetic lineages of constituent taxa, and the weighted approach accounts for relative abundances of constituent taxa. The taxa present in greater >5% mean relative abundance driving the distribution on the PCoA were *Corynebacterium*, *Staphylococcus*, *Pseudomonas*, *Prevotella*, and *Streptococcus*. There was a statistically significant relationship between stent material and composition on the weighted UniFrac analysis (Fig 2B; R^2^ = 0.09, p = 0.016; PERMANOVA;). In contrast, there was only a nonsignificant trend towards clustering by diagnosis (Fig 2A; R^2^ = 0.07, p = 0.06).

To examine whether alternative analytical approaches might change the nature of these results, we carried out the same analysis after raising the threshold for inclusion of an OTU to ≥10 reads across all samples (reducing the total to 3199 OTUs) and excluding OTUs that could potentially be derived from contamination sources [26] (leaving 3167 OTUs). We also applied rarefaction to 10,000 reads per sample. However, as shown in S3 Fig, this alternative analysis did not change the associations between biofilm diversity and clinical or stent features, nor impact the ordination on the PCoA plot. Thus, these findings are robust to different methodological approaches. Subsequent analyses therefore employed the more complete dataset.

When we examined taxa at the 1% mean relative abundance level (S4 Fig), *Corynebacterium* continued to define one dimension, suggesting a dominant effect on these communities, whereas multiple additional taxa contributed to other dimensions of the PCoA plot. We repeated this analysis without 10 specimens from one highly sampled subject, #0099, which revealed a similar pattern (S5 Fig), indicating that this relationship is not driven by subject oversampling.

Within the *Corynebacterium* genus there were 865 *de novo* OTUs with more than 10 reads assigned. However, >90% of reads were assigned to two OTUs, accounting for 71.8% and 18.9%, respectively. The top hits by BLASTn search of the NCBI 16S rRNA database for these OTUs were *C*. *striatum* (99% coverage and 100% identity) and *C*. *xerosis* (99% coverage and 99% identity), respectively; however, there is a high degree of homology in this region among *Corynebacterium*, and other species also had slightly lower but still >97% coverage and identity for these two OTU’s, thus precluding definitive assignment at the species level. Within the *Staphylococcus* genus there were 3 *de novo* OTUs that accounted for >95% of reads. The two most prevalent Staphylococcal OTUs could not be assigned at the genus level, while the top five BLASTn hits for the third most prevalent OTU (21% of *Staphylococcal* reads) were to *S*. *aureus* (100% coverage and 99% sequence identity), with other Staphylococcal species having lower coverage and/or identity.

### Temporal dynamics and subject-level factors influencing biofilms

We asked whether stent biofilms characteristics were linked to the duration that stents were in place. Duration of stent was not related to biofilm total bacterial biomass (S6 Fig; *r* = 0.18, p = 0.24), nor PCoA axes 1 or 2 (axis 1: *r* = 0.06, p = 0.69; axis 2: *r* = -0.01, p = 0.97). This suggests that factors other than stent duration determine community composition.

**Fig 3:**
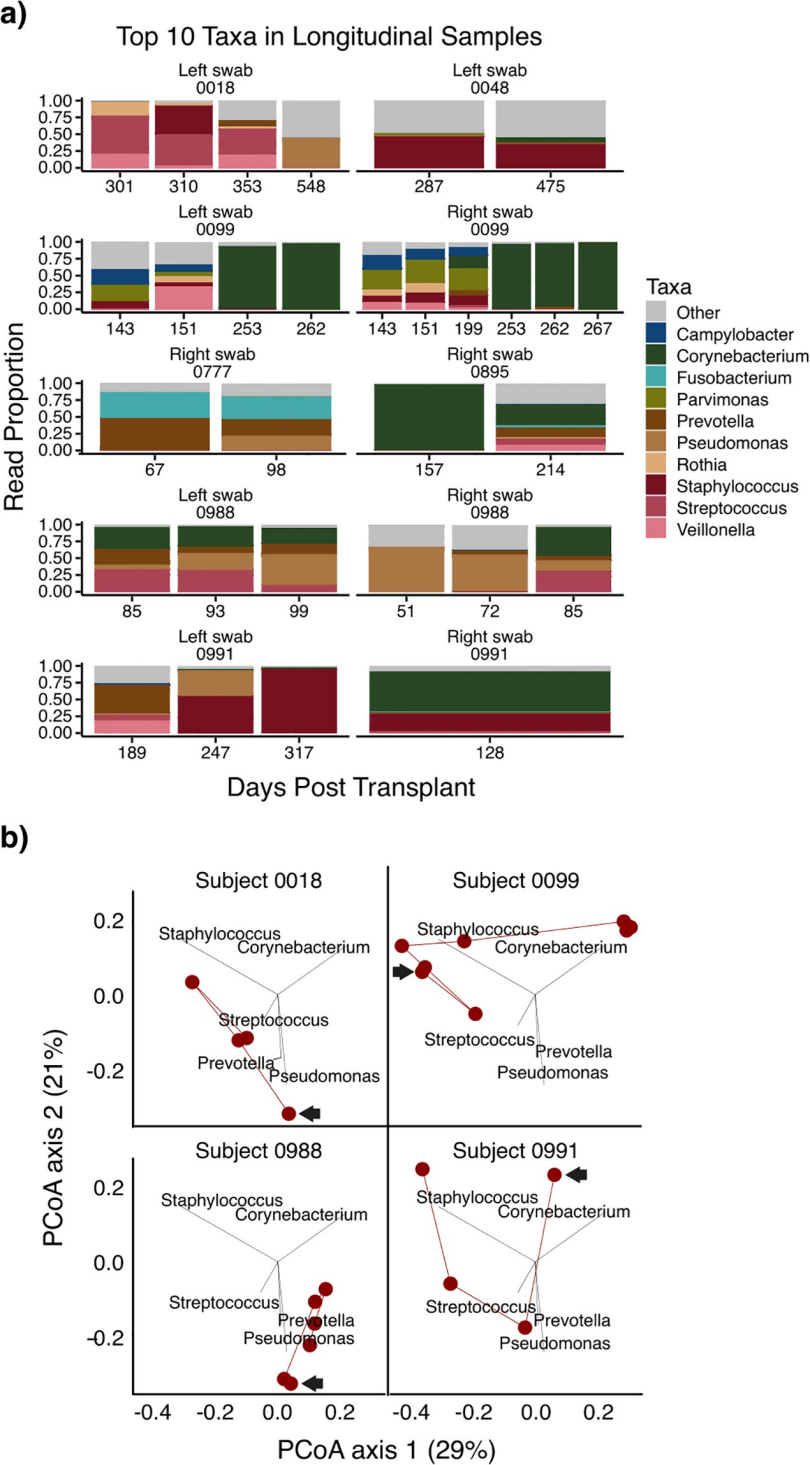
Change in stent biofilm composition over time in subjects with serial stents. (A) Biofilm communities from subjects with two or more stent samples; if bilateral stents were sampled, the right and left stents are shown separately. Stacked bar graphs represent the proportion of reads assigned the top ten most abundant bacterial genera across samples. The height of a segment of each bar is proportional to its relative abundance and its color reflects the assigned bacterial genus. The number below each sample indicates the number of days since lung transplant that the stent was removed. (B) Stent biofilm communities of four subjects who were sampled at three or more time points are shown on a weighted UniFrac PCoA of the entire sample set. The arrow indicates the first sample and the lines then connect samples sequentially. Three of the four subjects with longitudinal samples had stent biofilms that moved between different clusters over time.

Seven subjects had ≥2 stents available, including two with contemporaneous bilateral stents (Fig 3A). Sequential stents often showed consistent biofilm composition, but sometimes this was interrupted by gradual (#0991) or abrupt (#0099, #0988) compositional changes. In contrast, when bilateral stents were present, the communities were highly concordant (e.g. #0099 days 143, 151, 253, 262; #0988 day 85). We also visualized the 4 subjects with ≥3 stents on the PCoA plot (Fig 3B). Most showed considerable change. Together these results indicate that subject-level variables such as pre-transplant lung disease, and temporal factors such as time post-transplant or stent duration, did not have a primary role in determining stent biofilm composition.

### Identification of biofilm community types and their relationship to stent materials

**Fig 4:**
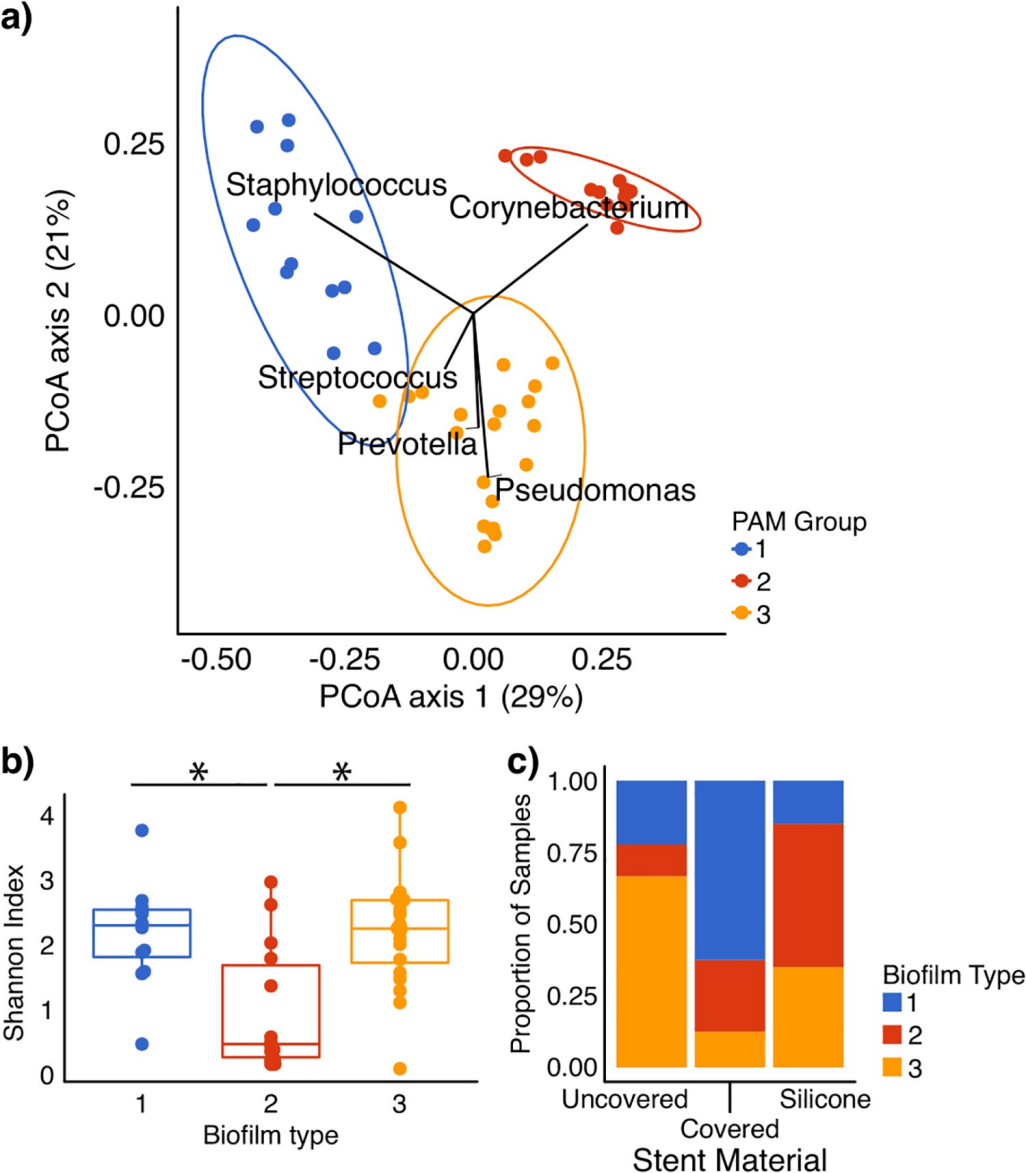
Partitioning about medoids (PAM) analysis of stent biofilm bacterial communities. (A) Weighted UniFrac PCoA, colored by biofilm type as determined by PAM analysis, analyzed at the genus level, which identified three groups with high confidence. Group 1 is driven primarily by *Staphylococcus*, Group 2 by *Corynebacterium*, and Group 3 by several bacteria including *Streptococcus*, *Prevotella*, and *Pseudomonas*. Ellipses indicate 95% confidence intervals for the data distribution within the categorical variable. (B) Shannon diversity of biofilm types defined by PAM. Biofilm type 2 (the Corynebacterium dominant type) had significantly lower diversity than the other types (p<0.05 for both, pairwise Wilcoxon test). (C) Stacked bar chart showing the proportion of biofilm samples of each biofilm type by stent material. Stent material was significantly associated with PAM group (p < 0.05, Fisher’ exact test and Chi Square Test). Silicone stents tended to have a higher proportion of *Corynebacterium*-dominated biofilm type 2, uncovered metal had more type 3, and covered metal had more type 1.

We applied two machine learning algorithms to investigate weighted UniFrac distance clustering: agglomerative hierarchical clustering (h-clust) and partitioning around medoids (PAM). Both found 3 clusters best explained the data (Fig 4). PAM biofilm type 1 was defined by *Staphylococcus*, type 2 by *Corynebacterium*, and type 3 by polymicrobial communities. Biofilm type 2 was clearly distinct, while types 1 and 3 reflected more of a continuum between community types (Fig 4A). The three PAM biofilm types had similar bacterial biomass based on 16S amplicon concentrations and were markedly greater than background (S1B Fig), with the Corynebacterium-dominated type 2 being slightly but non-significantly greater (p = 0.07, ANOVA). This result indicates that none of the biofilms reflect background or contamination.

We queried the taxa present above 1% mean relative abundance within each PAM biofilm type (Table 2). *Corynebacterium* was widely present. Furthermore, *Corynebacterium* dominated biofilm type 2, accounting for 86% of all reads. In contrast, PAM types 1 and 3 had more taxa above 1%. Concordantly, biofilm type 2 had lower diversity than the other types (Fig 4B).

**Table 2:** Top taxa within endobronchial stent biofilm PAM types.

Biofilm Type	> 1% Taxa	Proportion of Total Reads
**1**	Staphylococcus (g)	0.289
	Parvimonas (g)	0.119
	Campylobacter (g)	0.0957	
	Anaerococcus (g)	0.0859	
	Peptoniphilus (g)	0.0684	
	Streptococcus (g)	0.0594	
	Veillonella (g)	0.0547	
	Rothia (g)	0.0331	
	Pseudomonas (g)	0.0290	
	Oribacterium (g)	0.0266	
	Corynebacterium (g)	0.0246	
	Porphyromonas (g)	0.0243	
	Finegoldia (g)	0.0189	
	Bulleidia (g)	0.0185	
	Actinomyces (g)	0.0184	
**2**	Corynebacterium (g)	0.860	
	Anaerococcus (g)	0.0469	
	Actinomyces (g)	0.0241	
	Staphylococcus (g)	0.0141	
**3**	Pseudomonas (g)	0.162	
	Streptococcus (g)	0.146	
	Prevotella (g)	0.121	
	Corynebacterium (g)	0.106	
	Fusobacterium (g)	0.0745	
	Mycoplasma (g)	0.0744	
	Veillonella (g)	0.0568	
	Enterobacteriaceae (f)	0.0391	
	Actinomyces (g)	0.0267	
	Neisseriaceae (f)	0.0260	
	Rothia (g)	0.0209	
	Campylobacter (g)	0.0182	
	Neisseria (g)	0.0142	
	Parvimonas (g)	0.0133	
	Enterococcus (g)	0.0133	
	Anaerococcus (g)	0.0130	

Taxa reflecting >1% of reads across all stents within the biofilm type are listed, with their relative abundances, grouped at the genus (g) or family (f) level.

Stent material was significantly associated with biofilm type (Fig 4C; p = 0.008, Fisher’s exact test). Specifically, covered metal was associated with type 1 (*Staphylococcus*), silicone with type 2 (*Corynebacterium*), and uncovered metal with type 3 (polymicrobial). In contrast, biofilm type did not correlate with pre-transplant diagnosis.

### Stent biofilm relationship to the bronchoalveolar lavage (BAL) microbiome and environmental controls

Four subjects had BAL performed during the stent removal bronchoscopy. We compared the stent biofilm, BAL, and washes of the bronchoscope working channel prior to the procedure (S7 Fig). The stent biofilm and BAL bacterial composition were similar, and distinct from the environmental controls. In biofilms with high *Corynebacterium*, this bacterium was abundant in the BAL but not in environmental controls, indicating it was authentically present in the stent biofilm and lower respiratory tract, and not derived from environmental contamination.

### Fungal microbiome in stent biofilms

**Fig 5:**
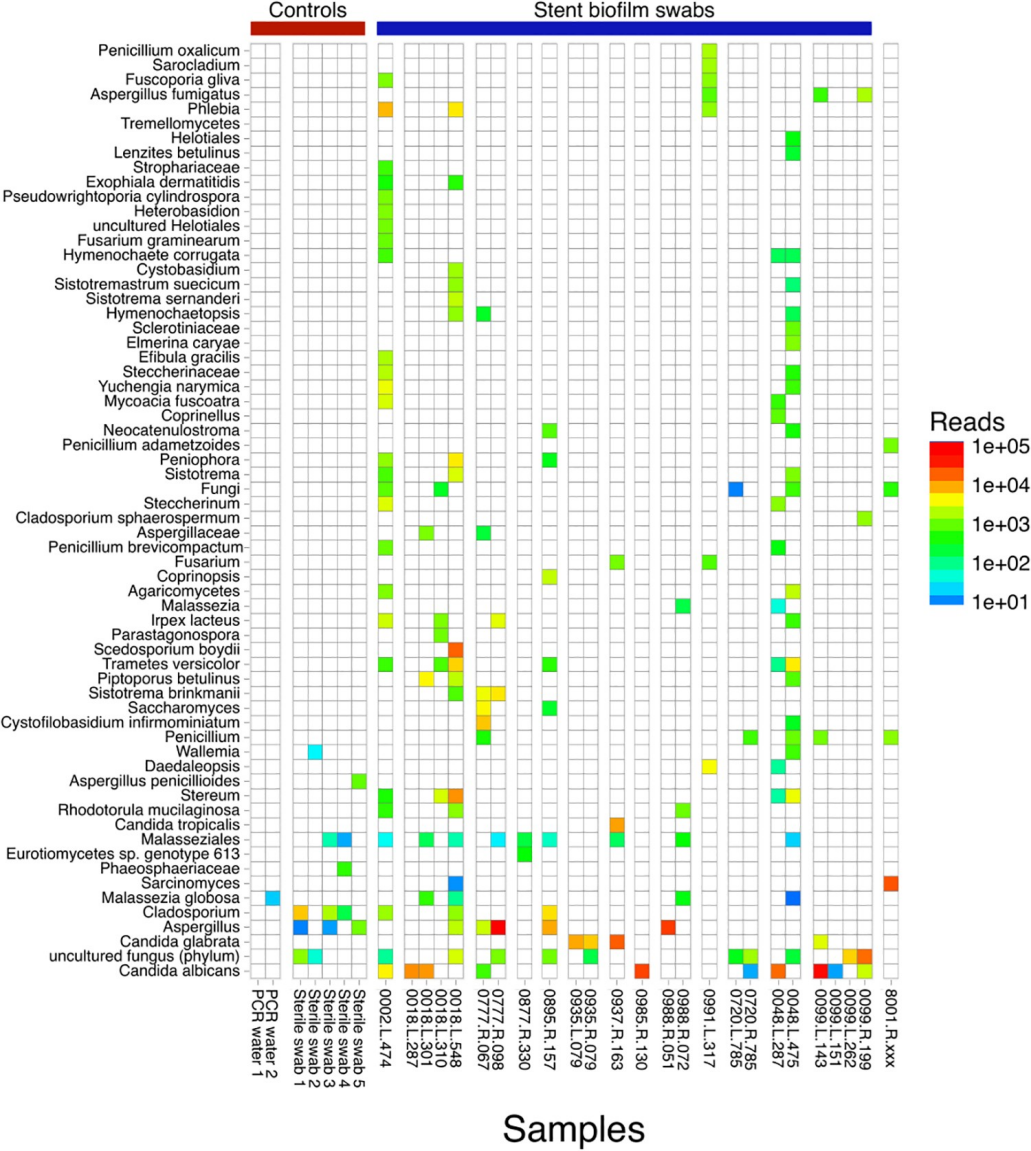
Fungal taxa within stent biofilms samples as determined by ITS sequencing. Twenty-five biofilm swab samples and seven control samples had fungal ITS amplification sufficient for sequencing. In the heat map, rows reflect fungal taxa at the lowest level assignment possible (down to the species level). Each column is a unique biofilm sample, grouped by subject. The name is in the format of: subject ID—side of stent—day post-transplant. Because the total fungal biomass is highly variable among samples, the color scale reflects absolute read counts rather than relative abundances to avoid exaggeration of taxa in low fungal content specimens. Taxa with fewer than 10 reads in any sample are excluded.

We used ITS sequencing to identify fungal taxa in the stent biofilm. Of the 46 specimens, 21 had no definitive fungal amplification using ITS primers and were not pooled for sequencing. We first visualized fungal taxa in the positive samples on a heat map (Fig 5). Because the presence of fungi is highly variable in lower respiratory tract specimens [22,27], we analyzed fungi by read count numbers rather than relative abundances, which can exaggerate the appearance of taxa in samples with few fungal reads [22].

Several stents yielded high numbers of ITS reads aligning to *Aspergillus* and *Candida*, and in some samples, *Scedosporium*, *Stereum*, and *Sarcinomyces* (Fig 5, Table 3). *Candida* species included *C*. *albicans*, *C*. *glabrata*, and *C*. *tropicalis*. Among samples with *Aspergillus*, three were confidently assigned to *A*. *fumigatus*, while the remainder could only be assigned to *Aspergillus* at the genus level. *Scedosporium* is occasionally recognized as an opportunistic pathogen. However, *Stereum* and *Sarcinomyces* were unexpected because they are typically considered environmental contaminants, yet they were present at high read numbers within biofilms and absent from our environmental controls. Environmental control specimens had very low numbers of fungal reads, suggesting that fungi identified at high read count in stent biofilms are authentic constituents rather than contamination-derived.

**Table 3:** Fungal reads identified in endobronchial stents.

Sample ID^A^	Taxonomic Assignment^B^^,^^C^	Read Count^C^
0002-L-474	Candida albicans (s)	5190
	Phlebia (g)	4982
	Yuchengia narymica (s)	4018
	Steccherinum (g)	3502
	Mycoacia fuscoatra (s)	3273
	Fungi (k)	3245
	Trametes gibbosa (s)	3075
	Irpex lacteus (s)	3005
0018-L-287	Candida albicans (s) *2 OTU’s	**12654**
0018-L-301	Candida albicans (s)	9824
	Piptoporus betulinus (s)	5023
0018-L-548	**Scedosporium boydii (s)**	**20098**
	**Stereum (g)**	**10012**
	Trametes versicolor (s)	7086
	Hypholoma sublateritium (s)	5106
	Trechispora stellulata (s)	4802
	Phlebia (g)	4245
	Peniophora (g)	3864
	Sisotrema (g)	3325
	Burgoa verzuoliana (s)	3232
	Phlebia radiate (s)	3038
0048-L-287	**Candida albicans (s)**	**17057**
0048-L-475	Trametes versicolor (s)	5510
	Stereum (g)	4070
0777-R-67	Cystofilobasidiium infirmominiatum (s)	7915
	Saccharomyces (g)	4956
	Sistotrema brinkmannii (s)	4166
0777-R-098	**Aspergillus (g)**	**111651**
	Sistotrema brinkmannii (s)	5813
	Irpex lacteus (s)	3707
8001-R-XXXX	**Sarcinomyces (g)**	**23361**
0895-R-157	Aspergillus (g)	7842
	Cladosporium (g)	4243
0935-L-079	**Candida glabrata (s)**	**10599**
0935-R-079	Candida glabrata (s)	7582
0937-R-163	**Candida glabrata (s)**	**21282**
	**Candida tropicalis (s)**	**11226**
0985-R-130	**Candida albicans (s)**	**24303**
0988-R-051	**Aspergillus (g)**	**29169**
0099-L-143	**Candida albicans (s)**	**41296**
	Candida glabrata (s)	3552
0099-L-262	Uncultured fungus (p)	6987
0099-R-199	**Uncultured fungus (p)**	**18322**
0991-L-317	Daedaleopsis (g)	4511
Sterileswab1	Cladosporium (g) *2 OTU’s	7817

Taxa with ≥3000 reads in a sample are shown, along with the number of reads.

^A^Sample ID corresponds to those of Fig 5.

^B^Taxa are shown at the most precise level that could be assigned, as species (s), genus (g), family (f), phylum (p), or in one case no better than kingdom (k).

^C^Taxa with greater than 10,000 reads in a sample are shown in bold.

In an analysis of bacterial/fungal co-variation, *Candida* showed a significant inverse correlation with the most abundant bacterial genus, *Corynebacterium* (S8 Fig; *ρ* = -0.48, p = 0.017, Spearman’s rank correlation). In contrast, there was no relationship between *Candida* and *Streptococcus* or other prevalent taxa.

### Relationship between biofilm composition, airway cultures, and clinical features

Ten subjects had airway cultures concurrent with stent removal; six cultures revealed respiratory pathogens (Table 1). In five out of the six positive cultures, the cultured bacteria were identified in the stent biofilm. Importantly, the respiratory pathogen was not the most abundant 16S rRNA sequence, but rather was part of a community that included anaerobic or upper respiratory-type bacteria that are not usually cultured. One subject (#0895) grew *Mycobacterium avium*-intracellulare (MAI), which was not identified by sequencing. This suggests that either MAI does not participate in stent biofilm communities even if present in the lower respiratory tract, or these molecular methods are less sensitive than culture for mycobacteria [27], even though our previous work suggests that this pipeline can detect mycobacteria [17]. Conversely, we detected *Enterococcus* by sequencing but not culture (#0099, day 267) five days after a prior positive airway culture for this organism.

Three subjects had stents placed for anastomotic dehiscence, and two of these had the highest numbers of *Aspergillus* reads in our study (#0777, #0988; Fig 5 and Table 3). Subject 0077 (day 98) had a contemporaneous fungal culture, which was positive for *Aspergillus*. *Candida* spp. were detected by sequencing in many stents, including several at high levels. *Candida* spp. are typically reported in respiratory cultures as yeast with no further identification by our clinical microbiology lab. The stents that had *Scedosporium*, *Stereum*, and *Sarcinomyces* did not have contemporaneous BAL cultures.

All but one subject was on at least one antibiotic at the time of stent removal and most were also exposed to anti- fungal medications (S1 Table). The ubiquity of the exposure and between-subject heterogeneity limits statistical analysis of the impact of specific antimicrobials on biofilm composition, but indicates that, overall, biofilm formation is robust to antimicrobial treatment. While the goal of this study was to understand the composition of luminal biofilms established, we also queried the relationship to clinical outcome, data for which was available in 13 subjects. We asked if biofilm type (limited to the final stent in subjects with >1 stent) was related to outcome categorized as resolution of the airway problem motivating stent placement, presence of excessive granulation tissue, and stent mucus plugging. Within these categories, there was no significant relationship between stent biofilm type and outcomes (p = 1, Fisher’s Exact Test), though the number of subjects in any individual outcome group was small in this cohort. Thus, the impact of biofilm types identified here on outcomes will require future prospective studies.

## Discussion

Here we report the first molecular investigation of the airway stent biofilm. In this predominantly post-transplant cohort, we identified three bacterial community types—one low diversity and dominated by *Corynebacterium*, while the other two have greater diversity and are characterized by *Staphylococcus* or by a mixed population including *Pseudomonas*, *Prevotella*, and *Streptococcus*. Biofilm type correlated with stent material. When individuals had serial stents, the biofilm communities frequently shifted, suggesting that device-related, procedural and/or contemporaneous clinical factors, but not pre-transplant lung disease, influence biofilm formation. Fungal sequences were frequently detected, sometimes at high read counts.

*Corynebacterium* was the most prevalent bacteria. It dominated biofilm type 2, and was present at substantial albeit lower abundance in other biofilm types. *Corynebacteria* are common skin flora and are generally not considered pathogens in the respiratory tract [6,28], although there have been reports of *C*. *striatum* respiratory tract infection in immunocompromised hosts[29–32]. One previous study identified *Corynebacterium* in airway cultures from 4.6% of patients after lung transplantation, and within this population the presence of airway stents was associated with Corynebacterium persistence [33]. Our data suggests a potentially important role for *Corynebacterium* is its interactions and competition with other bacteria and fungi in the biofilm. Supporting this notion, *Corynebacterium* spp. have been shown to antagonize *Staphylococcus aureus* and *Streptococcus pneumoniae* in the anterior nares [34–36]. Furthermore, *Corynebacterium* spp. have recently been identified as foundational taxa in dental biofilms, serving as a nidus of nucleation for other bacteria in dental plaque [11]. Thus, in the post-transplant stent biofilm *Corynebacterium* may be derived from the oral cavity and thrive within the biofilm, provide a scaffold for some taxa to persist while competing with others, and play a central role in endobronchial stent biofilm structure and composition.

Despite their importance for treating airway compromise, stents may themselves increase risk of lower respiratory tract complications, including infection, granulation tissue formation and mucous plugging [37,38]. Stent colonization has been postulated to contribute to these consequences [39–41], and so understanding biofilms that form on these devices could be important to optimizing their development and use. We found stent material was significantly correlated with biofilm type, with silicone stents favoring the *Corynebacterium*-dominated biofilms, covered metal favoring *Staphylococcus*-type biofilms, and uncovered metal associated with polymicrobial biofilms. Although the association was imperfect and explained only part of the biofilm composition, this information if further substantiated could help guide stent design or selection for the purposes of minimizing infectious and inflammatory complications. Such knowledge could be important in reducing the risk of colonization by potential pathogens. Future studies could address how antimicrobial-impregnated stent materials impact biofilm development.

Fungi were common within stent biofilms, with *Candida* being the most frequently identified, including pathogenic species *C*. *albicans*, *C*. *glabrata*, and *C*. *tropicalis*. While *Candida* are often considered contaminants in respiratory cultures, they have a propensity to establish biofilms [4,42,43]. Our observation of high read numbers of *Candida* spp. suggest they are bona fide inhabitants of these post-transplant airway stent biofilms. *Aspergillus* is often suspected as a cause of anastomotic complications requiring stent insertion [1], and we found a preponderance of *Aspergillus* reads in dehiscence cases. The presence of *Aspergillus* in these biofilms is not only consistent with such an association, but raises the possibility that the biofilm might also contribute to fungal persistence. Several other fungi were found in individual samples at high abundance, including *Scedosporium*, *Stereum*, and *Sarcinomyces*. *Scedosporium* is recognized as an opportunistic pathogen. In contrast, the latter two fungi are generally not considered human pathogens. Further study is needed to determine whether these biofilm inhabitants are linked to anastomotic tissue infections, either a cause or a consequence, or might perpetuate inflammation, impede healing, or cause stent/airway complications.

The fact that stent biofilms were markedly higher in bacterial biomass and differed in composition from environmental controls (S1 and S2 Figs) indicates that these findings authentically reflect biofilm and not contamination. In addition, the major findings are robust to different analytical approaches. Nevertheless, our study has several limitations. The study was observational so there was inherent heterogeneity in exposures, although accurately reflects clinical practice in complex patients. Sampling was done at the time of stent removal, so it is difficult to make inferences into the how the stent microbiome changed *in situ*. We investigated a relatively large number of stents (n = 46), but the number of any single stent material, underlying lung disease, and outcome group was modest. Finally, our study was done in a predominantly post-transplant population, which has distinct exposures to antibiotics and immunosuppression that may limit generalizability to stent biofilms that form in non-transplant populations.

In summary, this is the first study to comprehensive, unbiased molecular methods to interrogate the biofilm of airway stents. We identify a central role for *Corynebacterium* in the stent biofilm, three broad types of biofilm communities, evidence for unexpected uncultured fungi and fungal/bacterial community co-variation, a possible link between fungi and anastomotic dehiscence, and an association between stent material and biofilm type. Understanding the nature and composition of airway stent biofilms sets the stage for future studies to determine how luminal biofilms influence outcomes, identification of low-risk or high-risk biofilms and better understanding of mechanisms involved in their establishment. This knowledge will offer opportunities for improved stent technology and decision-making around stent selection and management.

## Supporting information

S1 File Supplementary methods. (DOCX)

S1 Fig Boxplot of comparing microbial biomass between sample types. (A) Stent swabs have a higher biomass using 16S amplicon quantification compared to bronchoscope pre-wash and lab controls (p < 0.001 for both comparisons); (B) PAM groups are not statistically different in biomass (p = 0.23 comparing groups 1–2 and 2–3, p = 0.48 comparing 1–3).
(TIF)

S2 Fig Heatmap of bacterial taxa identified across all sample types. Each row reflects a taxon with greater than 2000 reads assigned to it across samples. Each column is a sample with an annotation above noting its sample type and the 16S amplicon quantification through PicoGreen.
(TIF)

S3 Fig Sensitivity analyses of alpha and beta diversity indices using alternative filtering approaches. Diversity analyses were repeated after filtering OTUs with less than 10 reads across samples, filtering OTUs flagged by the *decontam* package, and rarefaction to of samples to 10,000 reads. (A, B) Shannon diversity remained significantly lower in stent swabs from subjects with underlying suppurative disease compared to COPD (p = 0.0033) and ILD (p = 0.021). (C, D) Biofilm composition remains significantly related to stent material (PERMANOVA, R^2^ = 0.09, p = 0.019) but not diagnosis (R^2^ = 0.07, p = 0.09) by weighted UniFrac.
(TIF)

S4 Fig Principal coordinate analysis of stent microbial communities. Weighted UniFrac PCoA analysis of stent biofilm communities showing vectors representing the genus-level bacteria present above 1% relative abundance that explain the ordination on the PCoA. Panel (A) is colored by diagnosis and panel (B) is colored by stent material.
(TIF)

S5 Fig Principal coordinate analysis after removal of an oversampled subject. Weighted UniFrac PCoA of stent biofilm samples, excluding 10 samples from subject 0099. Vectors show bacterial taxa driving the ordination that are present at greater than 5% relative abundance. After removal of the oversampled subject’s data, similar clusters form and are driven by the same bacterial genera as in the full dataset, suggesting that this subject’s samples did not disproportionately skew community clustering.
(TIF)

S6 Fig Analysis of biomass and PAM groups as a function of time. Biofilm samples were grouped based on the duration stent was in place prior to removal: from 0 to 30 days, 31 to 60 days, and greater than 60 days. Panel (A) shows the relationship between stent duration and bacterial biomass as assessed by 16S amplicon quantity, and panel (B) proportion of samples belonging to each biofilm PAM group.
(TIF)

S7 Fig Comparison of taxa in stent biofilm and contemporaneous BAL. Four subjects had bronchoalveolar lavage (BAL) carried out contemporaneous with stent removal, with samples shown as heatmaps, along with matched bronchoscope pre-wash samples as a background control. Each column is a sample, each row is a bacterial taxon, and each group represents a different subject. The left-hand panel shows the number of matched reads per sample; because the environmental controls are low biomass samples we used absolute read counts rather than relative abundances so taxa with low numbers of reads within a low microbial biomass sample are not exaggerated. The right-hand panel shows the relative abundances within each sample. Within each subject grouping, the first column is a BAL sample (B), second is a stent biofilm (S), and third is a bronchoscope pre-wash environmental control (P).
(TIF)

S8 Fig Fungal-bacterial covariation. For all samples with at least 500 fungal reads (n = 22), the absolute reads on a log10 scale of *Candida* within the ITS dataset (x-axis) were plotted against the proportion *Corynebacterium* and *Streptococcus* (y-axis). The line is fit with a general linear regression model with 95% confidence intervals shown in gray. There was a significant inverse relationship between *Candida* and *Corynebacterium* (*ρ* = -0.48, p = 0.017, Spearman’s rank correlation) but no correlation with *Streptococcus* relative abundance. The regression line is derived from a generalized linear regression model with 95% confidence interval in gray.
(TIF)

S1 Table Anti-microbial exposure at the time of stent removal. (DOCX)

## Abbreviations

16S rRNA gene: 16S ribosomal ribonucleic acid gene
BAL: bronchoalveolar lavage
CF: cystic fibrosis
COPD: chronic obstructive pulmonary disease
hclust: hierarchical clustering
ILD: interstitial lung disease
IPF: idiopathic pulmonary fibrosis
ITS: internal transcribed spacer
PAM: partitioning around medoids
PCoA: principal coordinate analysis
QIIME: quantitative insights into microbial ecology
V1-V2: hypervariable regions 1 & 2 of the 16S rRNA gene
