# Supplementary material for: Molecular analysis of the endobronchial stent microbial biofilm reveals bacterial communities that associate with stent material and frequent fungal constituents
Source: PLoS One. 2019 May 29;14(5):e0217306. doi: 10.1371/journal.pone.0217306 (PMC6541290; doi:10.1371/journal.pone.0217306)
Supplement: S1 File — (DOCX) [file pone.0217306.s001.docx]

Molecular analysis of the endobronchial stent microbial biofilm reveals bacterial communities that associate with stent material and frequent fungal constituents

John E. McGinniss^1¶^, Ize Imai^1¶^, Aurea Simon-Soro^1^, Melanie C. Brown^1^, Vincent R. Knecht^1^, Laura Frye^1^, Priyanka M. Ravindran^1^, Marisol I. Dothard^1^, Dylan A. Wadell^1^, Michael B. Sohn^3^, Hongzhe Li^3^, Jason D. Christie^1, 3^, Joshua M. Diamond^1^, Andrew R. Haas^1^, Anthony R. Lanfranco^1^, David M. DiBardino^1^, Frederic D. Bushman^2^*, Ronald G. Collman^1,2^*

^1^Department of Medicine, Division of Pulmonary, Allergy and Critical Care, Perelman School of Medicine at the University of Pennsylvania, Philadelphia, Pennsylvania, United States of America

^2^Department of Microbiology, Perelman School of Medicine at the University of Pennsylvania, Philadelphia, Pennsylvania, United States of America

^3^Department of Epidemiology, Biostatistics and Informatics, Perelman School of Medicine at the University of Pennsylvania, Philadelphia, Pennsylvania, United States of America

¶ These authors contributed equally to this work

**Supplemental Methods**

**DNA extraction and purification**

DNA was extracted from stent swabs using the PowerSoil DNA isolation kit (MoBio, Carlsbad, CA) following the manufacturer’s protocol except for additional bead beating step after chemical lysis with the Mini-Beadbeater-16 (BioSpec, Bartlesville, OK) at 3450 oscillations/sec in a ‘figure 8’ direction for 2 minutes along with a 95^o^C incubation to improve DNA recovery from fungi. Extractions were done on an individual basis with one sample per tube. All extractions were done in a BSL2+ hood after the workspace was decontaminated with DNA-remover and UV irradiation.

**PCR amplification and sequencing**

Bacterial 16S rRNA gene amplification was done with V1V2 primers (forward primer 27F (5'- AGAGTTTGATCCTGGCTCAG-3') and reverse primer 338R (5'-TGCTGCCTCCCGTAGGAGT-3')) and using PCR conditions previously described[1,2]. We had 13,771,660 raw sequences after MiSeq sequencing and then 6,881,764 after quality filtering

Fungal ITS sequencing was done by amplifying the fungal ITS1 region with ITSF1 (5’-CTTGGTCATTTAGAGGAAGTAA-3') and ITS2 (5’-GCTGCGTTCTTCATCGATGC-3') dual-barcoded primers as previously described[1,2]. Clinical samples and controls were PCR amplified in three replicates with each replicate dual-barcoded with unique Golay primer pairs. PCR reactions contain 4 µl of DNA template, 16.1 µl of PCR grade water, 0.4 µl of AccuPrime Taq polymerase High Fidelity (5U/µl; Invitrogen), 2.5 µl of Buffer II and each primer at 30 µM. Thermocycling involved initial denaturation at 95°C for 5 minutes, followed by 30 cycles of 95°C for 30 sec, 56°C for 30 sec and 72°C for 90 sec, and ending with extension at 72°C for 8 minutes. The three replicates of individual samples were combined before cleaning with 0.8x bead to 1x PCR product volume ratio with AgenCourt AMPure XP Magnetic beads (Beckman Coulter) per manufacturer protocol. Samples that did not have discernable amplification as measured using the Agilent 22000 TapeStation system (Agilent Technologies, Santa Clara, CA) with High Sensitivity D1000 ScreenTape, or amplification was far below that of primer dimers, were not included in subsequent library preparation. Remaining samples were pooled at equimolar amounts, as well as 20 µl each of PCR amplicons for controls. Libraries were prepared with MiSeq Reagent Kit v2 (300-cycles, Illumina). The number of input sequences were 1,017,188 and 759,607 after ITS quality filtering.

**Fungal ITS sequence processing**

Fungal sequences were processed with PIPITS (v1.3.4, full documentation at: https://github.com/hsgweon/pipits) and annotation done with BROCC (https://github.com/kylebittinger/brocc) [3,4]. The PIPITS pipeline joins paired-ends with PEAR (https://cme.h-its.org/exelixis/web/software/pear/doc.html#cl-usage), which by default requires a minimum overlap of 10bp and performs statistical testing to minimize false positive read pairing (p<0.01 threshold). PIPITS then performs quality filtering (filters chimeras, removes reads <100bp), uses ITSx through HMMER3 Hidden Markov Models to extract ITS sequences, and then creates an OTU abundance table, which by default clusters at 97% sequence identity. We assigned taxonomy by taking the output representative sequences from PIPITS and inputting them into BROCC. This pipeline generates consensus-based taxonomic assignments from BLAST results of the input sequences.

**References:**

1. Charlson ES, Diamond JM, Bittinger K, Fitzgerald AS, Yadav A, Haas AR, et al. Lung-enriched organisms and aberrant bacterial and fungal respiratory microbiota after lung transplant. Am J Respir Crit Care Med. 2012;186: 536–545. doi:10.1164/rccm.201204-0693OC

2. Clarke EL, Lauder AP, Hofstaedter CE, Hwang Y, Fitzgerald AS, Imai I, et al. Microbial lineages in sarcoidosis a metagenomic analysis tailored for low-microbial content samples. Am J Respir Crit Care Med. 2018;197: 225–234. doi:10.1164/rccm.201705-0891OC

3. Gweon HS, Oliver A, Taylor J, Booth T, Gibbs M, Read DS, et al. PIPITS: An automated pipeline for analyses of fungal internal transcribed spacer sequences from the Illumina sequencing platform. Methods Ecol Evol. 2015;6: 973–980. doi:10.1111/2041-210X.12399

4. Dollive S, Peterfreund GL, Sherrill-Mix S, Bittinger K, Sinha R, Hoffmann C, et al. A tool kit for quantifying eukaryotic rRNA gene sequences from human microbiome samples. Genome Biol. 2012;13: R60. doi:10.1186/gb-2012-13-7-r60
