# Supplementary figures and images for: Molecular analysis of the endobronchial stent microbial biofilm reveals bacterial communities that associate with stent material and frequent fungal constituents
Source: PLoS One. 2019 May 29;14(5):e0217306. doi: 10.1371/journal.pone.0217306 (PMC6541290; doi:10.1371/journal.pone.0217306)

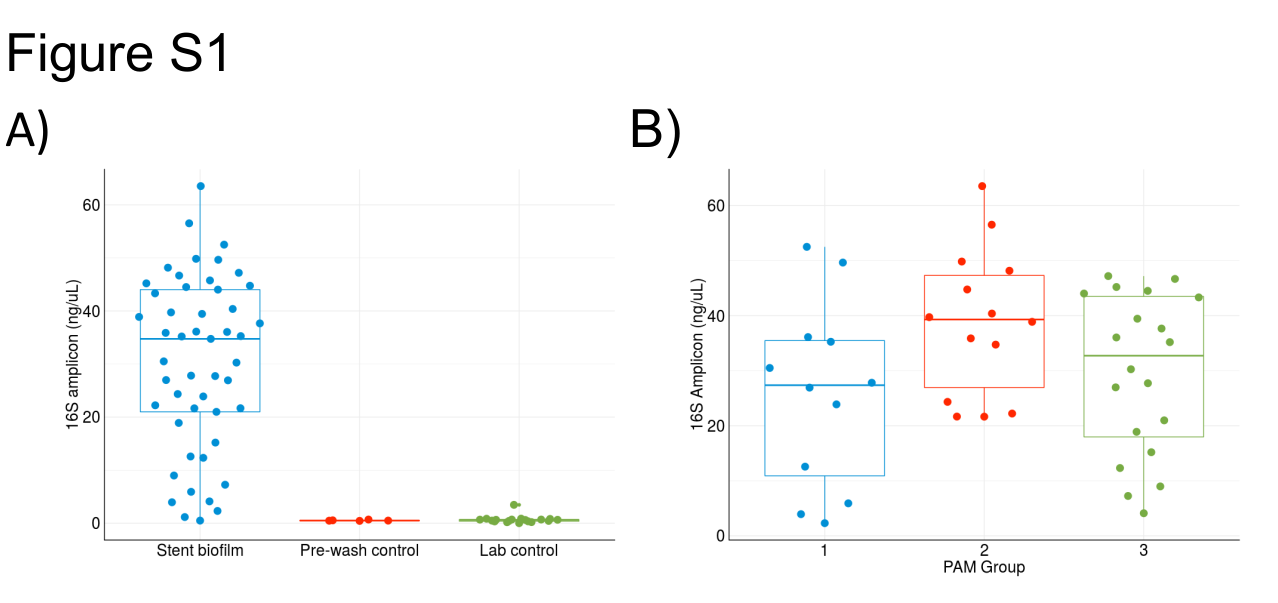

Supplement: S1 Fig — (A) Stent swabs have a higher biomass using 16S amplicon quantification compared to bronchoscope pre-wash and lab controls (p < 0.001 for both comparisons); (B) PAM groups are not statistically different in biomass (p = 0.23 comparing groups 1–2 and 2–3, p = 0.48 comparing 1–3). (TIF) [file pone.0217306.s002.tif]

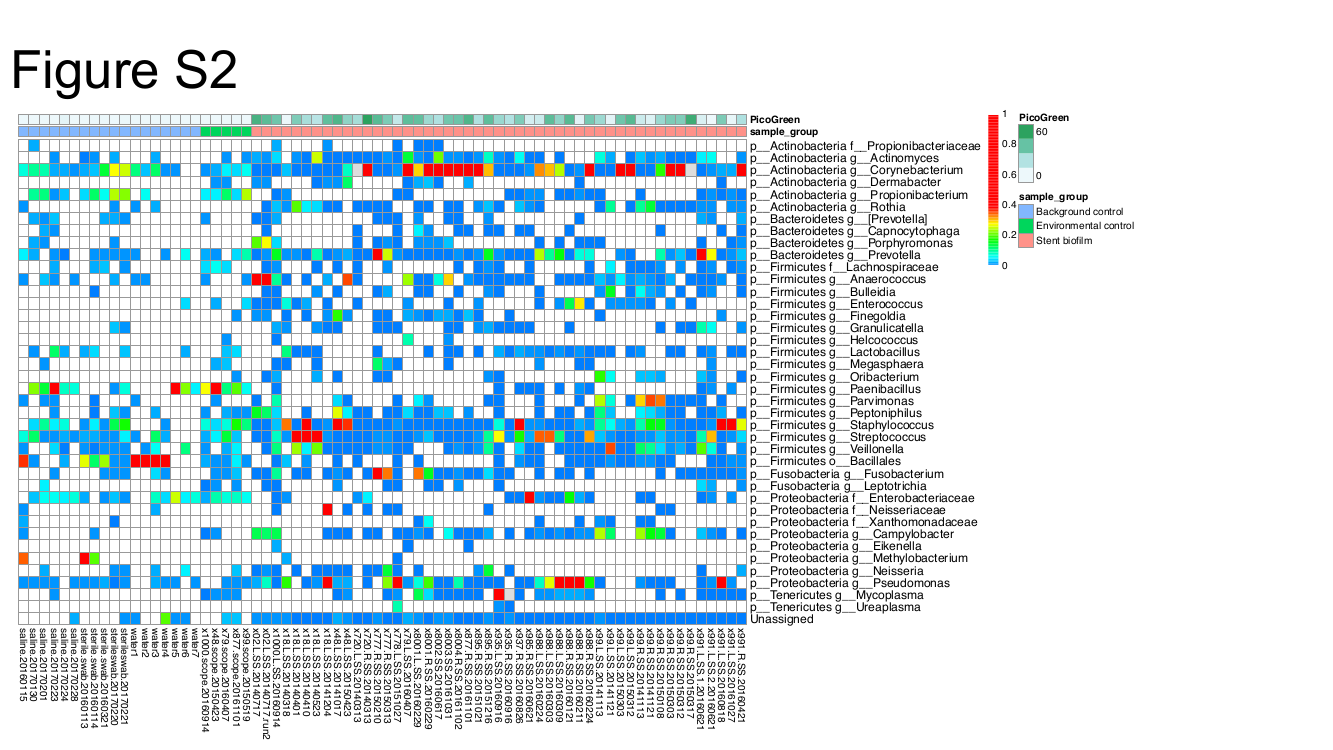

Supplement: S2 Fig — Each row reflects a taxon with greater than 2000 reads assigned to it across samples. Each column is a sample with an annotation above noting its sample type and the 16S amplicon quantification through PicoGreen. (TIF) [file pone.0217306.s003.tif]

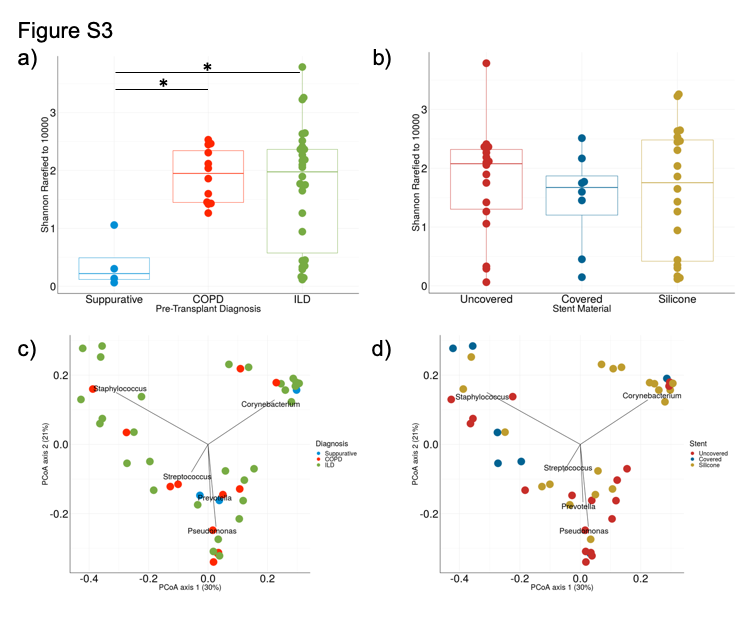

Supplement: S3 Fig — Diversity analyses were repeated after filtering OTUs with less than 10 reads across samples, filtering OTUs flagged by the decontam package, and rarefaction to of samples to 10,000 reads. (A, B) Shannon diversity remained significantly lower in stent swabs from subjects with underlying suppurative disease compared to COPD (p = 0.0033) and ILD (p = 0.021). (C, D) Biofilm composition remains significantly related to stent material (PERMANOVA, R2 = 0.09, p = 0.019) but not diagnosis (R2 = 0.07, p = 0.09) by weighted UniFrac. (TIF) [file pone.0217306.s004.tif]

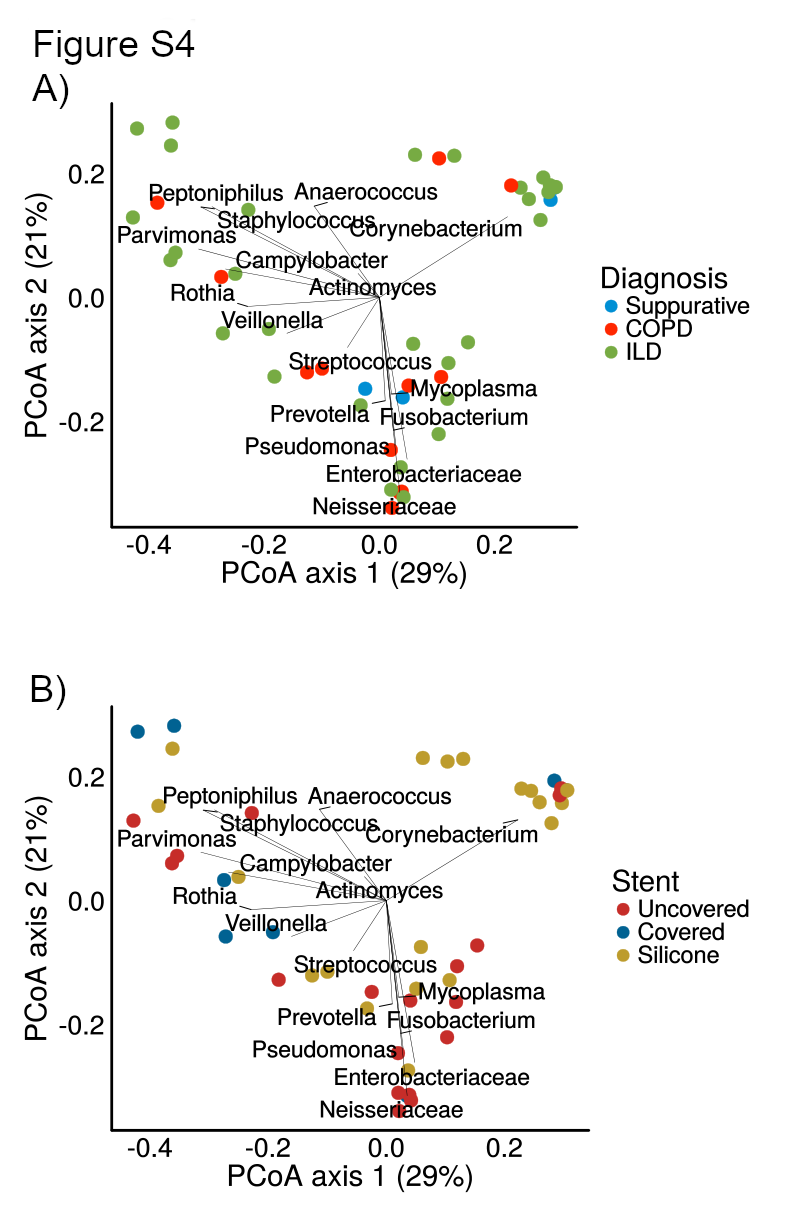

Supplement: S4 Fig — Weighted UniFrac PCoA analysis of stent biofilm communities showing vectors representing the genus-level bacteria present above 1% relative abundance that explain the ordination on the PCoA. Panel (A) is colored by diagnosis and panel (B) is colored by stent material. (TIF) [file pone.0217306.s005.tif]

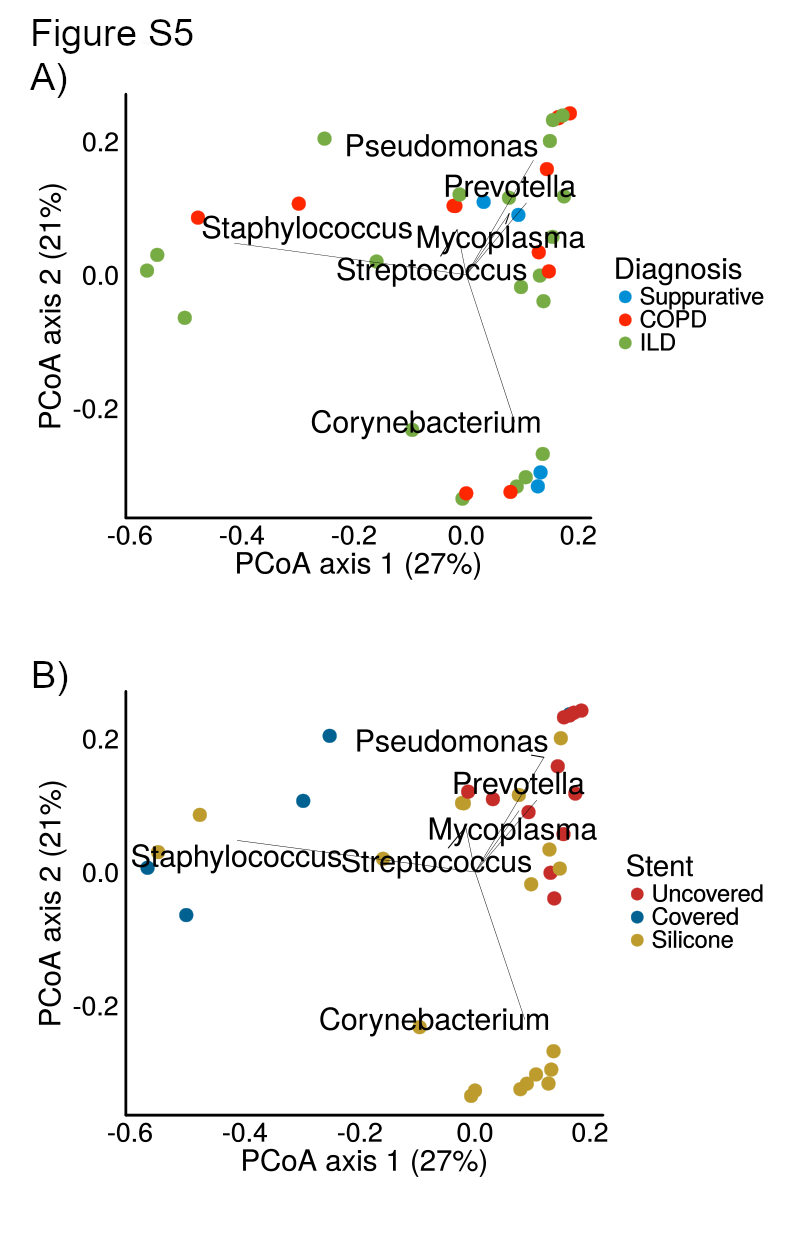

Supplement: S5 Fig — Weighted UniFrac PCoA of stent biofilm samples, excluding 10 samples from subject 0099. Vectors show bacterial taxa driving the ordination that are present at greater than 5% relative abundance. After removal of the oversampled subject’s data, similar clusters form and are driven by the same bacterial genera as in the full dataset, suggesting that this subject’s samples did not disproportionately skew community clustering. (TIF) [file pone.0217306.s006.tif]

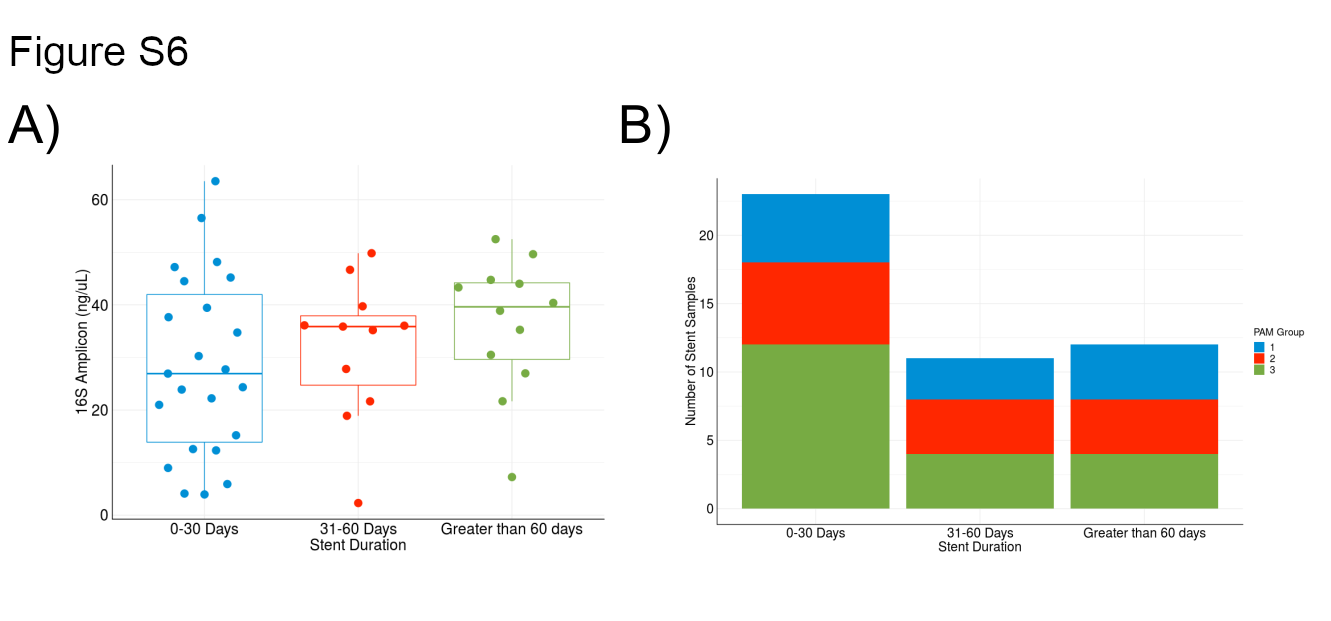

Supplement: S6 Fig — Biofilm samples were grouped based on the duration stent was in place prior to removal: from 0 to 30 days, 31 to 60 days, and greater than 60 days. Panel (A) shows the relationship between stent duration and bacterial biomass as assessed by 16S amplicon quantity, and panel (B) proportion of samples belonging to each biofilm PAM group. (TIF) [file pone.0217306.s007.tif]

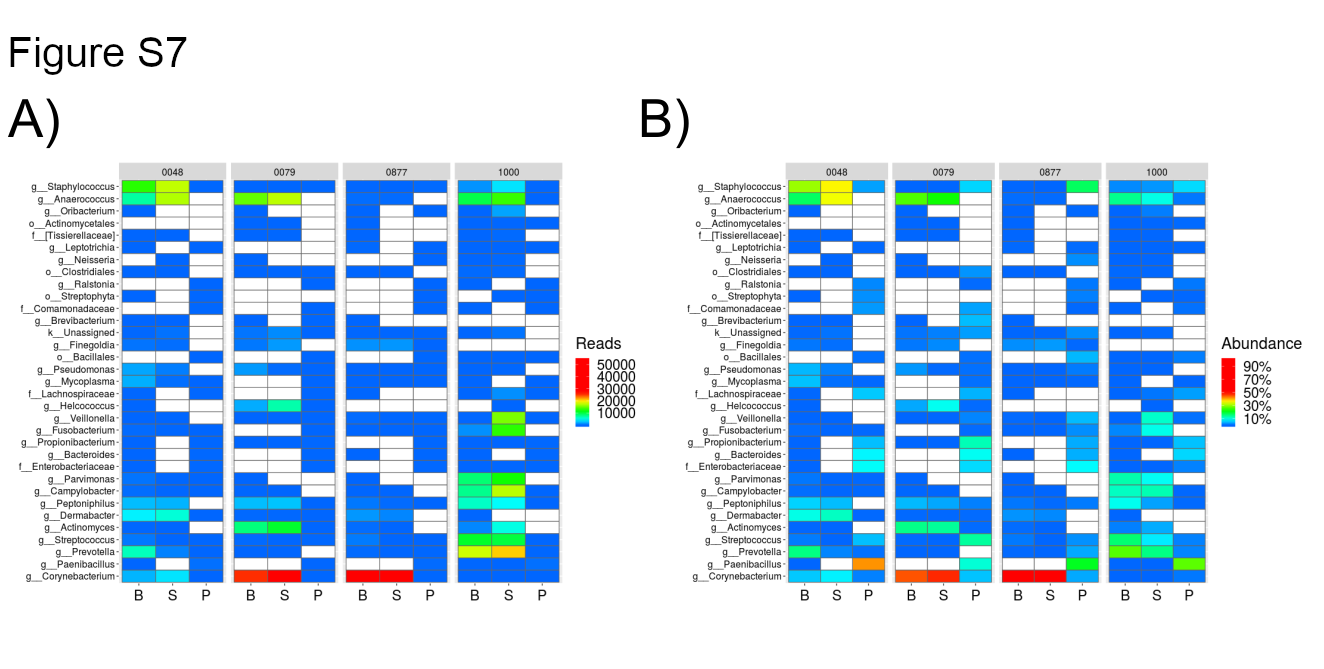

Supplement: S7 Fig — Four subjects had bronchoalveolar lavage (BAL) carried out contemporaneous with stent removal, with samples shown as heatmaps, along with matched bronchoscope pre-wash samples as a background control. Each column is a sample, each row is a bacterial taxon, and each group represents a different subject. The left-hand panel shows the number of matched reads per sample; because the environmental controls are low biomass samples we used absolute read counts rather than relative abundances so taxa with low numbers of reads within a low microbial biomass sample are not exaggerated. The right-hand panel shows the relative abundances within each sample. Within each subject grouping, the first column is a BAL sample (B), second is a stent biofilm (S), and third is a bronchoscope pre-wash environmental control (P). (TIF) [file pone.0217306.s008.tif]

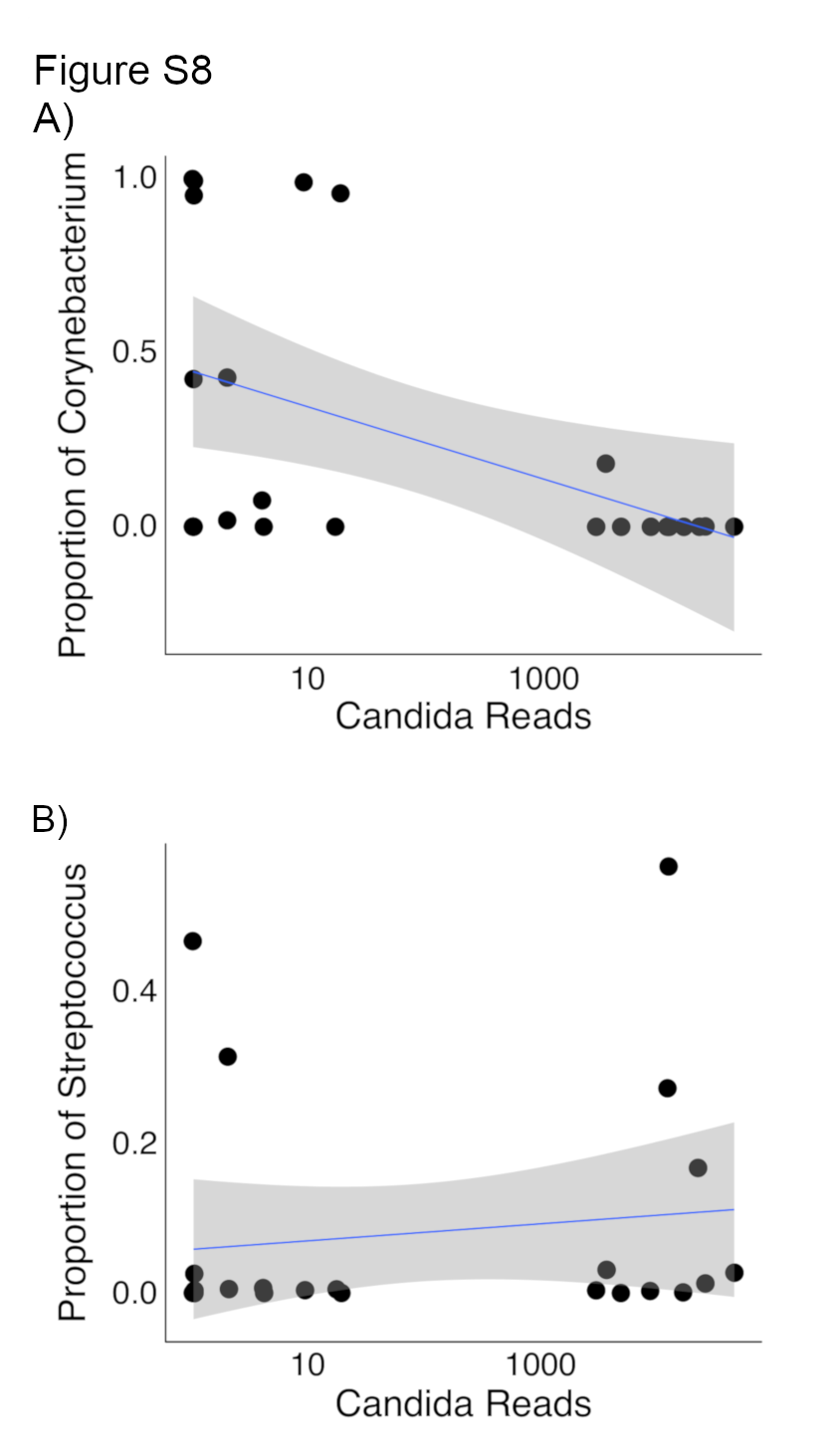

Supplement: S8 Fig — For all samples with at least 500 fungal reads (n = 22), the absolute reads on a log10 scale of Candida within the ITS dataset (x-axis) were plotted against the proportion Corynebacterium and Streptococcus (y-axis). The line is fit with a general linear regression model with 95% confidence intervals shown in gray. There was a significant inverse relationship between Candida and Corynebacterium (ρ = -0.48, p = 0.017, Spearman’s rank correlation) but no correlation with Streptococcus relative abundance. The regression line is derived from a generalized linear regression model with 95% confidence interval in gray. (TIF) [file pone.0217306.s009.tif]
