# Supplementary material for: Molecular analysis of the endobronchial stent microbial biofilm reveals bacterial communities that associate with stent material and frequent fungal constituents
Source: PLoS One. 2019 May 29;14(5):e0217306. doi: 10.1371/journal.pone.0217306 (PMC6541290; doi:10.1371/journal.pone.0217306)
Supplement: S1 Table — (DOCX) [file pone.0217306.s010.docx]

**S1 Table.** **Anti-microbial exposure at the time of stent removal.**

| **Subject** | **Post-Transplant**  **Day** | **Antibacterial** | **Antiviral** | **Antifungal** |
| --- | --- | --- | --- | --- |
| **0002** | 474 | Azithromycin, Cephalexin, Bactrim | Valganciclovir | NA |
| **0018** | 301 | Azithromycin, Bactrim, Tobramycin | Valacyclovir | Voriconazole |
|  | 310 | Azithromycin, Bactrim, Tobramycin | Valacyclovir | Voriconazole |
|  | 353 | Azithromycin, Bactrim, Tobramycin | Valacyclovir | Voriconazole |
|  | 548 | Azithromycin, Bactrim, Tobramycin | Valganciclovir | Voriconazole |
| **0079** | 454 | Bactrim | NA | Nystatin |
| **0720** | 785 | Azithromycin, Bactrim, Clindamycin, Tobramycin | Valganciclovir | Nystatin |
| **0777** | 67 | Bactrim | Valganciclovir | Amphotericin B, Voriconazole |
|  | 98 | Bactrim | Valganciclovir | Amphotericin B, Voriconazole |
| **0778** | 20 | NA | NA | NA |
| **0877** | 330 | Azithromycin, Levofloxacin | NA | Atovaquone |
| **0895** | 157 | Bactrim, Ertapenem | Valganciclovir | Voriconazole |
|  | 214 | Bactrim | Valganciclovir | Voriconazole |
| **0935** | 79 | NA | Valganciclovir | Atovaquone, Voriconazole |
| **0937** | 163 | Bactrim, Tobramycin | Valganciclovir | Posaconazole |
| **0985** | 130 | Bactrim, Vancomycin | Valganciclovir | Nystatin, Voriconazole |
| **0988** | 51 | Bactrim, Tobramycin | Valganciclovir | NA |
|  | 72 | Bactrim, Tobramycin | Valganciclovir | NA |
|  | 85 | Bactrim, Tobramycin | Valganciclovir | NA |
|  | 93 | Bactrim, Tobramycin | Valganciclovir | NA |
|  | 99 | Bactrim, Tobramycin | Valganciclovir | NA |
| **0991** | 128 | Bactrim | Valganciclovir | Amphotericin, Posaconazole |
|  | 189 | Doxycycline, Bactrim, Levofloxacin | Valganciclovir | Amphotericin |
|  | 247 | Bactrim | NA | Amphotericin |
|  | 317 | Bactrim, Tobramycin, Levofloxacin | Valganciclovir | Amphotericin |
| **1000** | 98 | Amoxicillin, Bactrim, Cefpodoxime | Valganciclovir | NA |
| **8001** | 1932 | Amoxicillin, Bactrim | Valacyclovir | NA |
| **8002** | NA | NA | NA | NA |
| **8003** | 649 | Azithromycin, Bactrim, Cephalexin | NA | NA |
| **8004** | 979 | Azithromycin, Bactrim | NA | Nystatin |
| **0048** | 287 | Bactrim, Levofloxacin | Valacyclovir | Itraconazole |
|  | 475 | Amoxicillin, Levofloxacin, Bactrim, Tobramycin | Valacyclovir | NA |
| **0099** | 143 | Bactrim, Levofloxacin, Tobramycin | Valacyclovir | Voriconazole |
|  | 151 | Bactrim, Levofloxacin, Tobramycin | Valacyclovir | Voriconazole |
|  | 199 | Levofloxacin, Bactrim, Tobramycin | Valacyclovir | Amphotericin, Voriconazole |
|  | 253 | Bactrim | Valacyclovir | Amphotericin, Voriconazole |
|  | 262 | Bactrim | Valacyclovir | Amphotericin, Voriconazole |
|  | 267 | Bactrim | Valacyclovir | Voriconazole |
